# Lysophosphatidic Acid Signaling Axis Mediates Ceramide 1-Phosphate-Induced Proliferation of C2C12 Myoblasts

**DOI:** 10.3390/ijms19010139

**Published:** 2018-01-04

**Authors:** Caterina Bernacchioni, Francesca Cencetti, Alberto Ouro, Marina Bruno, Antonio Gomez-Muñoz, Chiara Donati, Paola Bruni

**Affiliations:** 1 Department of Experimental and Clinical Biomedical Sciences “Mario Serio”, University of Florence, Viale GB Morgagni 50, 50134 Firenze, Italy; caterina.bernacchioni@unifi.it (C.B.); francesca.cencetti@unifi.it (F.C.); marina.bruno@stud.unifi.it (M.B.); paola.bruni@unifi.it (P.B.); 2 Istituto Interuniversitario di Miologia (IIM), Italy; 3 Department of Biochemistry and Molecular Biology, Faculty of Science and Technology, University of the Basque Country (UPV/EHU), 48080 Bilbao, Spain; alber.ouro@gmail.com (A.O.); antonio.gomez@ehu.es (A.G.-M.); 4 Department of Developmental Biology and Cancer Research, The Institute for Medical Research Israel-Canada, The Hebrew University-Hadassah Medical School, Jerusalem 91120, Israel

**Keywords:** lysophosphatidic acid (LPA), ceramide 1-phosphate (C1P), lysophosphatidic acid receptor (LPAR), skeletal muscle, myoblast proliferation

## Abstract

Sphingolipids are not only crucial for membrane architecture but act as critical regulators of cell functions. The bioactive sphingolipid ceramide 1-phosphate (C1P), generated by the action of ceramide kinase, has been reported to stimulate cell proliferation, cell migration and to regulate inflammatory responses via activation of different signaling pathways. We have previously shown that skeletal muscle is a tissue target for C1P since the phosphosphingolipid plays a positive role in myoblast proliferation implying a role in muscle regeneration. Skeletal muscle displays strong capacity of regeneration thanks to the presence of quiescent adult stem cells called satellite cells that upon trauma enter into the cell cycle and start proliferating. However, at present, the exact molecular mechanism by which C1P triggers its mitogenic effect in myoblasts is lacking. Here, we report for the first time that C1P stimulates C2C12 myoblast proliferation via lysophosphatidic acid (LPA) signaling axis. Indeed, C1P subsequently to phospholipase A2 activation leads to LPA_1_ and LPA_3_ engagement, which in turn drive Akt (protein kinase B) and ERK1/2 (extracellular signal-regulated kinases 1/2) activation, thus stimulating DNA synthesis. The present findings shed new light on the key role of bioactive sphingolipids in skeletal muscle and provide further support to the notion that these pleiotropic molecules might be useful therapeutic targets for skeletal muscle regeneration.

## 1. Introduction

Sphingolipids are not only crucial for membrane architecture but act as critical regulators of cell functions and are involved in different pathological conditions [1,2]. The “sphingolipid rheostat” was proposed more than 20 years ago; according to it, the levels of ceramide and sphingosine 1-phosphate (S1P), two interconvertible sphingolipid metabolites, determine the cell fate mediating opposite signaling pathways [3]. Ceramide has indeed been demonstrated to induce cell growth arrest and cell death [4]; on the other side, S1P has been linked to proliferation and survival [5]. Recently, to adequately address the complex role of sphingolipid metabolism in the regulation of cell behavior, the S1P/ceramide rheostat has been reconsidered also considering other sphingolipid species and the precise localized production and secretion of these metabolites that may participate in the regulation of cell fate [6]. For example, the fact that many of the effects of S1P are mediated by its ligation to S1P receptors (S1PR) has added nuances to the model. S1P intracellularly generated by the enzyme sphingosine kinase (SK), is released by the action of different S1P transporters in the extracellular environment and, in an autocrine/paracrine manner, binds to five differentially expressed G protein-coupled receptors, S1PR (S1P_1–5_), by the so-called “inside-out” signaling [7]. Moreover, ceramide can be phosphorylated by the action of ceramide kinase (Cerk) to generate another bioactive sphingolipid named ceramide 1-phosphate (C1P). Cerk, the only enzyme so far identified to be responsible for C1P biosynthesis in mammals, is localized in the cytosol, in the nucleus, in the Golgi apparatus as well as at the plasma membrane. C1P has been reported to stimulate cell proliferation of different cell types via activation of different signaling pathways such as ERK1/2 (extracellular signal-regulated kinases 1/2), JNK (c-Jun N-terminal kinases), PKCα (protein kinase C-α), Akt (protein kinase B) and mTOR (mammalian target of rapamycin) [8,9,10,11,12,13]. C1P is also a potent regulator of cell migration and of inflammatory responses via direct or PKC-mediated activation of cytosolic phospholipase A2 (cPLA2) [14,15,16,17,18,19]. Moreover, it has been demonstrated that C1P plays a role in the inhibition of apoptosis mainly by inhibiting ceramide formation. Plasma concentrations of C1P can vary up to 20 µM [20], being released mainly by macrophages and damaged cells [16,21].

Many experimental findings support a crucial role for sphingolipid signaling in the regeneration of skeletal muscle [22,23]. Skeletal muscle is a tissue composed of myofibers, post-mitotic cells that derive from the fusion of cells named myoblasts. Skeletal muscle displays a strong capacity of regeneration thanks to the presence of resident muscle stem cells called satellite cells. This heterogeneous population of adult stem cells is quiescent in uninjured muscle and becomes active upon a trauma that induces its proliferation and differentiation. Appropriate activation of satellite cells is fundamental to ensure a correct regenerative process. Upon injury, satellite cells in their niche are exposed to extrinsic proliferative cues such as growth factors and cytokines secreted by muscle fibers and other non-myogenic cells. The local microenvironment where satellite cells reside deeply changes with aging and skeletal muscle pathology [24,25]. Understanding the precise molecular mechanisms of action of skeletal muscle activating cues and their misregulation in aging and disease will highlight possible pharmacological targets to improve the cure of currently incurable skeletal muscle pathologies.

S1P has been reported to mediate the entry of satellite cells into the cell cycle, suggesting that the degradation of sphingomyelin observed upon cell activation leads to S1P production [26,27]. In addition, stimulation of cultured C2C12 myoblasts with S1P enhanced their differentiation into myotubes via S1P_2_ [28] and the over-expression of SK1 isoform in murine myoblasts enhanced their differentiation [29]. S1P has been involved in the modulation of the biological effects of cytokines, such as TNFα (tumor necrosis factor-α) and TGFβ (transforming growth factor-β) and growth factors, such as IGF-1 (insuline-like growth factor-1) and PDGF (platelet-derived growth factor) [30,31,32,33]. Moreover, we reported that lysophosphatidic acid (LPA), a potent bioactive lysophospholipid that acts mainly via engagement of multiple G protein-coupled receptors (LPAR), significantly enhanced migration of activated satellite cells through a cross-talk with SK and S1PR [34]. On the contrary, ceramide appears to be negatively associated with myoblast differentiation [35]; indeed, when its synthesis was blocked, the differentiation of rat myoblasts was enhanced [36].

We previously showed that skeletal muscle is a tissue target for C1P since the phosphosphingolipid plays a positive role in myoblast proliferation implying a role in muscle regeneration [9]. However, the exact molecular mechanism involved in the stimulation of proliferation by C1P is still lacking. Here, we report for the first time that C1P stimulates C2C12 myoblast proliferation via LPA signaling axis. Indeed, C1P subsequently to PLA2 activation leads to LPA_1_ and LPA_3_ engagement which in turn drives Akt and ERK1/2 activation thus stimulating DNA synthesis.

## 2. Results

**Figure 1:**
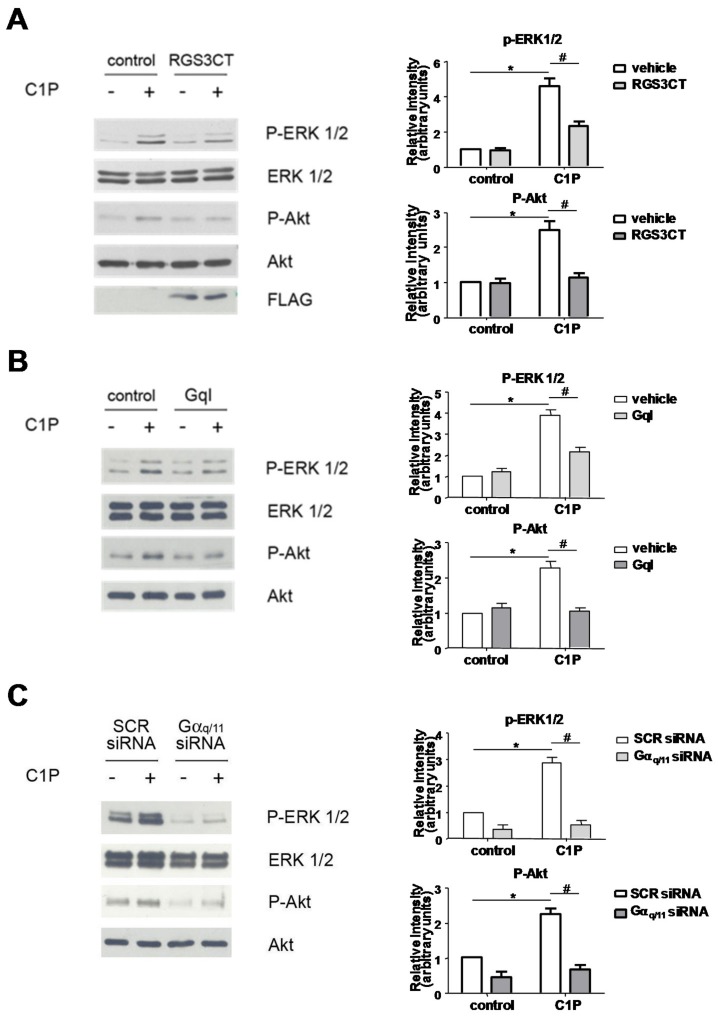
C1P signaling relies on Gαq/11 engagement. C2C12 cells were transfected with pcDNA3.1-RGS3CT tagged with: FLAG-tag M2 epitope or empty vector (**A**); transiently transfected with pRK5-GqI or empty vector (**B**); or with scrambled (SCR)- or Gαq/11-siRNA (**C**). Cells were overnight serum-starved prior to be stimulated with 15 µM C1P for 5 min. Left panels: ERK1/2 and Akt activation was measured in total cell lysates by Western blotting analysis using anti-phospho-ERK1/2 and anti-phospho-Akt antibodies. In (**A**), the efficiency of transfection was checked by WB analysis of the FLAG-tag. A blot representative of three independent experiments is shown. Right panels: Band intensity was quantified by densitometric analysis and normalized to the expression of total ERK1/2 and total Akt, respectively. C1P activates ERK1/2 and Akt in a statistically significant manner by Student’s *t* test (* *p* < 0.05); the effect of Gαq/11 down-regulation/inhibition was statistically significant by two-way ANOVA followed by Bonferroni’s post hoc test (^#^ *p* < 0.05).

We previously demonstrated that C1P induces proliferation of C2C12 myoblasts through the activation of phosphatidylinositol 3-kinase/Akt and ERK1/2 signaling pathways [9]. Moreover, in the same study, the mitogenic effect of the sphingolipid was found to be independent from the Gi protein-coupled receptor specific for C1P, which instead mediated the migration of RAW 264.7 macrophages [15]. To characterize the molecular mechanism involved in the proliferative action of C1P in C2C12 myoblasts, we examined whether C1P-induced activation of ERK1/2 and Akt could be mediated by another G protein-coupled receptor. For this purpose, myoblasts were transiently transfected with a plasmid encoding the N-terminally truncated version of Regulators of G protein Signaling (RGS)-3 (RGS3CT) that acts as GTPase-activating protein for the Gαi (Gα adenylyl cyclase inhibitor) and Gαq/11 (G protein subunit that activates phospholipase C) subfamilies [37,38]. The inactivation of Gαi and Gαq/11 significantly reduced the phosphorylation of ERK1/2 and Akt induced by 5 min treatment with 15 µM C1P measured by Western blot analysis on myoblast lysates (Figure 1A).

These data point to a major role for Gαq/11 in the signal transduction mechanism triggered by C1P. In accordance, the overexpression of a carboxyl-terminal peptide of Gαq that inhibits Gq-mediated signaling (GqI) [39], significantly decreased C1P-induced activation of ERK1/2 and Akt (Figure 1B). The RGS3CT and GqI efficiency in inhibiting Gq downstream signaling in myoblasts has been preliminarily verified in experiments where the membrane translocation of PKCα induced by bradykinin, known to act via Gq-coupled receptor [40,41], was abrogated by the overexpression of RGS3CT and GqI (Figure S1). The dependence of C1P signaling upon the engagement of a Gq-coupled receptor was further proved by the specific down-regulation of Gαq/11 through RNA interference: data shown in Figure 1C clearly show that the C1P-dependent activation of ERK1/2 and Akt was strongly diminished by Gαq/11 specific down-regulation.

**Figure 2:**
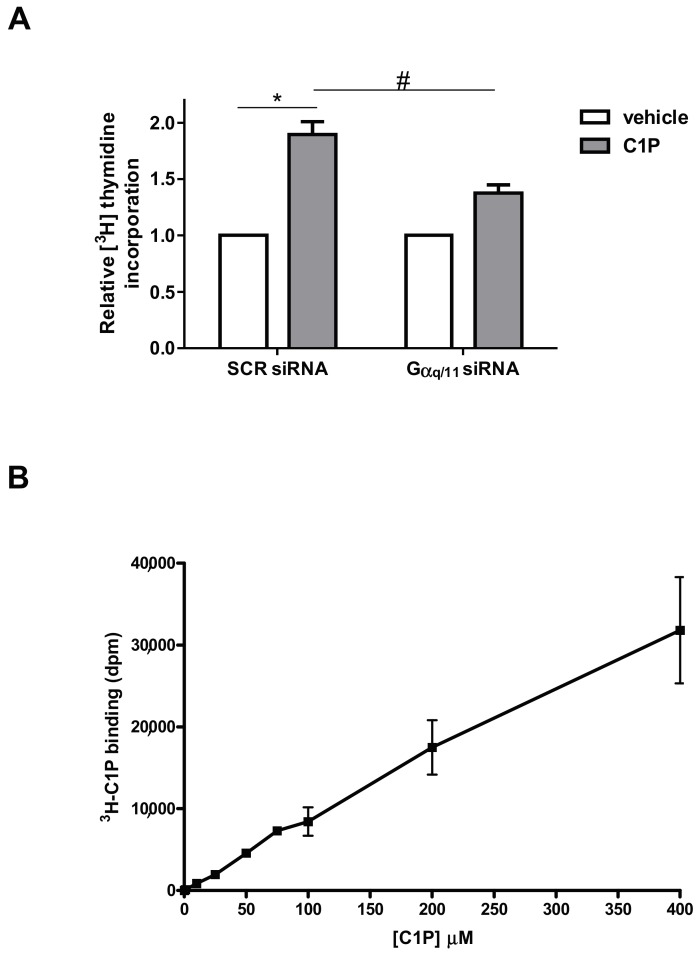
The mitogenic effect of C1P relies on Gαq/11 engagement. (**A**) Scrambled (SCR-) or specific Gαq/11-siRNA transfected C2C12 cells were treated or not with 15 µM C1P for 16 h. [^3^H]Thymidine (0.5 μCi/well) was added during the last hour of incubation. Results are reported as fold change over the control set as 1. Data are mean ± SEM of at least three independent experiments performed in triplicate. [^3^H]Thymidine incorporation in SCR-siRNA transfected control cells was 7398 ± 14 dpm, in Gaq/11 siRNA transfected cells was 1854.33 ± 487.28 dpm. C1P induces myoblast proliferation in a statistically significant manner by Student’s *t* test (* *p* < 0.05); the effect of Gαq/11 down-regulation was statistically significant by two-way ANOVA followed by Bonferroni’s post hoc test (^#^ *p* < 0.05). (**B**) Competitive assay of specific binding of [^3^H]C1P to myoblast cell membranes with different concentrations of C1P. Freshly prepared membranes were incubated with 10 μM [^3^H]C1P in the presence of the indicated concentrations of unlabeled C1P at 37 °C with gentle mixing for 30 min, in a total volume of 150 μL, in borosilicate tubes. Non-specific binding was measured with 10 μM [^3^H]C1P in the presence of the indicated concentrations of unlabeled C1P in absence of myoblast membranes at 37 °C with gentle mixing for 30 min, in a total volume of 150 μL, in borosilicate tubes. Results are the mean ± SEM of four independent experiments performed in triplicate. Radioactivity of filter-bound radionuclide was quantified by liquid scintillation counting.

Notably, as depicted in Figure 2A, specific down-regulation of Gαq/11 significantly affected C1P-induced-[^3^H]Thymidine incorporation into DNA, demonstrating that the engagement of a Gαq/11-coupled receptor mediates, at least in part, the mitogenic action of C1P in myoblasts. To characterize the putative receptor involved we performed radio-ligand binding assay. However, as shown in Figure 2B, specific ^3^H-labeled C1P binding to cell membranes was concentration-dependent and did not display a saturation kinetic up to 400 µM C1P. These findings thus excluded the engagement of a specific C1P receptor in the regulation of proliferation by C1P in C2C12 myoblasts.

**Figure 3:**
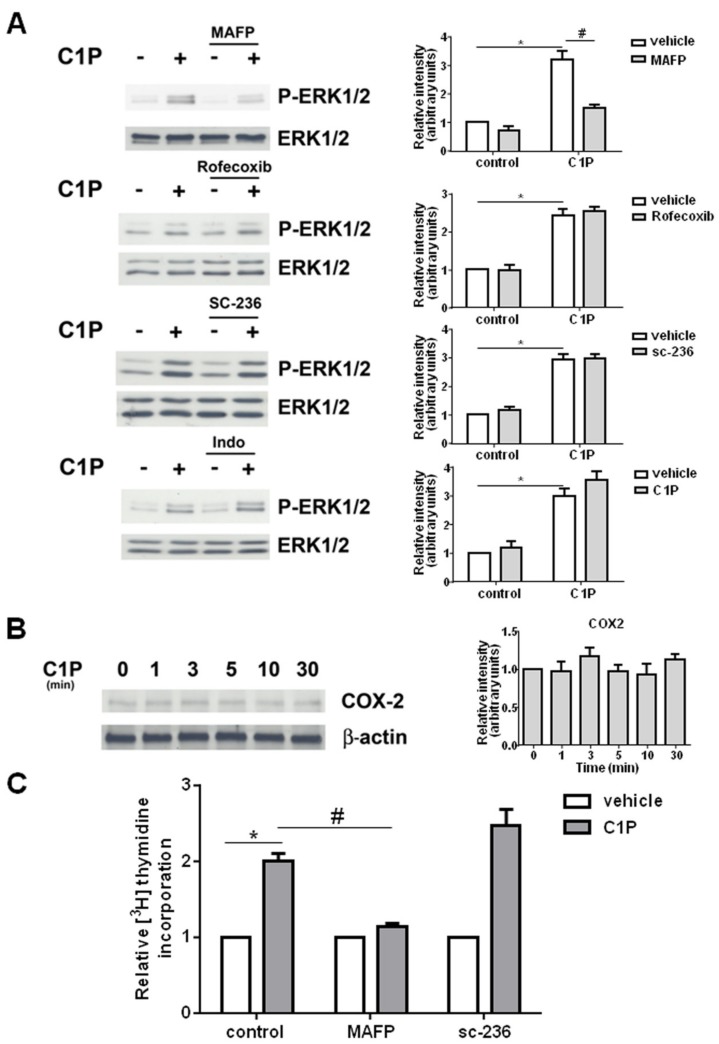
The mitogenic effect of C1P requires PLA2 but not COX2 activation. (**A**) Serum-starved C2C12 cells were pre-incubated for 30 min in the presence or absence of 25 μM MAFP or 100 μM Rofecoxib or 10 μM SC-236 or 50 μM Indomethacin (Indo), before being stimulated with 15 μM C1P for 5 min. ERK1/2 activation was measured in total cell lysates by Western blotting analysis using anti-phospho-ERK1/2 antibody. A blot representative of three independent experiments is shown. Right panels: Band intensity was quantified by densitometric analysis and normalized to the expression of total ERK1/2. C1P activates ERK1/2 in a statistically significant manner by by Student’s *t* test (* *p* < 0.05); the effect of PLA2 inhibition by MAFP, and not by Rofecoxib or SC-236 or Indomethacin, was statistically significant by two-way ANOVA followed by Bonferroni’s post hoc test (^#^ *p* < 0.05). (**B**) Western blot analysis was performed using specific anti-COX2 antibody in cell lysates prepared from serum-starved myoblasts treated with 15 μM C1P for the indicated time intervals. A blot representative of at least three independent experiments with analogous results is shown. Right panel: Band intensity was quantified by densitometric analysis and normalized to the expression of β-actin. (**C**) Serum-starved C2C12 myoblasts were pre-incubated for 30 min in the presence or absence of 25 μM MAFP or 10 μM SC-236 before being stimulated with 15 μM C1P for 16 h. [^3^H]Thymidine (0.5 μCi/well) was added during the last hour of incubation. Data are mean ± SEM of at least three independent experiments performed in triplicate. [^3^H]Thymidine incorporation in untreated control cells was 12,322.33 ± 2691.68 dpm, in MAFP-treated cells was 10,112.33 ± 916.04 dpm, and in SC-236-treated cells was 2905.3 ± 379.44 dpm. The mitogenic effect of C1P was statistically significant by Student’s *t* test (* *p* < 0.05); the effect of PLA2 inhibition by MAFP, but not by sc-236, was statistically significant by two-way ANOVA followed by Bonferroni’s post hoc test (^#^ *p* < 0.05).

It has been reported that C1P is able to bind and activate cPLA2 both in vitro and in vivo [42,43]. To gain insight into the mechanism by which C1P stimulates myoblast proliferation, we examined whether the phosphosphingolipid could elicit its biological effect through the activation of cPLA2 that catalyzes the hydrolysis of the sn-2 position of membrane glycerophospholipids thus releasing arachidonic acid (AA), the precursor of eicosanoids. To examine the possible role of PLA2 in C1P-mediated signaling, WB analysis of ERK1/2 phosphorylation was performed in C2C12 myoblasts previously incubated in the presence of the cPLA2 selective inhibitor, MAFP (methyl arachidonyl fluorophosphonate, 25 μM). As illustrated in Figure 3A, inhibition of cPLA2 strongly decreased the activation of ERK1/2 induced by C1P suggesting that this enzyme is required for C1P-mediated activation of this signaling pathway in myoblasts.

It was then investigated whether cyclooxygenase (COX), the enzyme responsible for the conversion of cPLA2-generated AA into prostanoids, was involved in C1P-mediated biological effect in myoblasts. For this purpose, both the constitutive COX1 and the inducible COX2 isoforms were blocked using specific pharmacological inhibitors. As depicted in Figure 3A, the specific inhibition of COX2 with 100 μM Rofecoxib or 10 μM SC-236 did not affect ERK1/2 phosphorylation induced by C1P. Similarly, pre-treatment with the COX inhibitor Indomethacin (50 μM) did not alter C1P-induced ERK1/2 phosphorylation (Figure 3A). Analogous results were obtained with increasing concentrations up to 200 μM of Indomethacin. Accordingly, Western blot analysis demonstrated that 15 μM C1P was unable to modulate the expression of COX2 at least up to 30 min of incubation (Figure 3B). These findings suggest that COX-induced prostanoid generation was not involved in C1P-induced ERK1/2 activation in myoblasts. In agreement with these data, 25 μM MAFP blunted the enhancement of [^3^H]Thymidine incorporation into DNA elicited by C1P while 10 μM SC-2365 did not affect the mitogenic effect of the bioactive sphingolipid (Figure 3C). Therefore, while the inhibition of cPLA2 significantly reduced the incorporation of radioactive thymidine into DNA induced by the sphingolipid, COX2 inhibition was inefficacious.

Since PLA2 action on phosphatidic acid, besides generating AA, releases another crucial pleiotropic bioactive lipid, namely LPA, known as potent inducer of cell proliferation in different cell types via interaction with its specific LPAR, we checked whether this bioactive lysolipid might represent the possible mediator of the mitogenic action of C1P in myoblasts.

**Figure 4:**
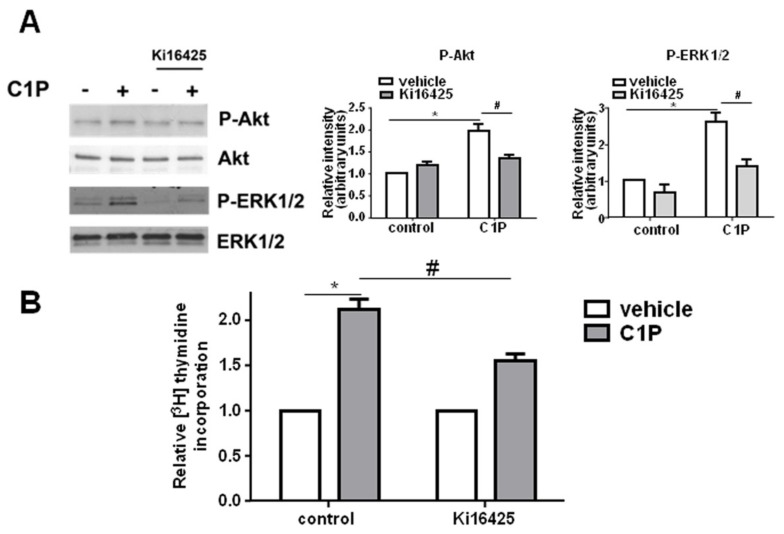

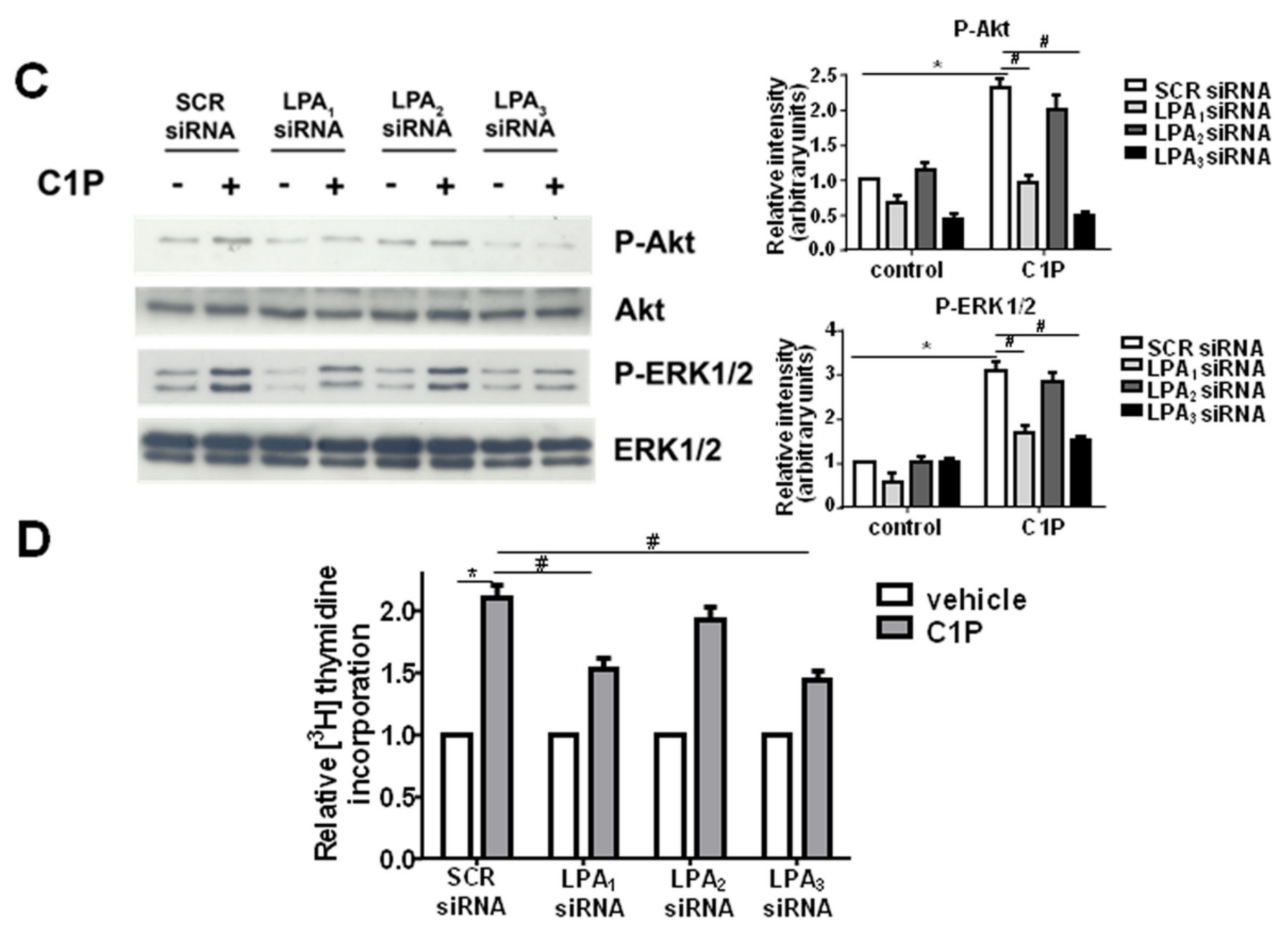
The mitogenic effect of C1P relies on LPA_1_ and LPA_3_ engagement. (**A**) Overnight serum-starved C2C12 myoblasts were pre-incubated for 30 min in the presence or absence of 2.5 μM Ki16425 before being stimulated with 15 μM C1P for 5 min. Left panel: ERK1/2 and Akt activation was measured in total cell lysates by Western blot analysis using anti-phospho-ERK1/2 and anti-phospho-Akt antibodies. A blot representative of three independent experiments is shown. Right panels: Band intensity was quantified by densitometric analysis and normalized to the expression of total ERK1/2 and total Akt, respectively. C1P activates ERK1/2 and Akt in a statistically significant manner by Student’s *t* test (* *p* < 0.05); the effect of LPA_1/3_ inhibition by Ki16425 was statistically significant by two-way ANOVA followed by Bonferroni’s post hoc test (^#^ *p* < 0.05). (**B**) Serum-starved C2C12 myoblasts were pre-incubated for 30 min in the presence or absence of 2.5 μM Ki16425 before being stimulated with 15 μM C1P for 16 h. [^3^H]Thymidine (0.5 μCi/well) was added during the last hour of incubation. Data are mean ± SEM of at least three independent experiments performed in triplicate. [^3^H]Thymidine incorporation in untreated control cells was 14,820.5 ± 891.36 dpm, and in Ki16425 treated cells was 8959 ± 512 dpm. The mitogenic effect of C1P was statistically significant by Student’s *t* test (* *p* < 0.05); the effect of LPA_1/3_ inhibition by Ki16425 was statistically significant by two-way ANOVA followed by Bonferroni’s post hoc test (^#^ *p* < 0.05). (**C**) C2C12 cells transfected with scrambled (SCR-) or with specific siRNA for individual LPA receptors were serum-starved prior to be stimulated with 15 µM C1P for 5 min. Left panels: ERK1/2 and Akt activation was measured in total cell lysates by Western blot analysis using anti-phospho-ERK1/2 and anti-phospho-Akt antibodies. A blot representative of three independent experiments is shown. Right panels: Band intensity was quantified by densitometric analysis and normalized to the expression of total ERK1/2 and total Akt, respectively. C1P activates ERK1/2 and Akt in a statistically significant manner by Student’s *t* test (* *p* < 0.05); the effect of LPA_1_ or LPA_3_ down-regulation was statistically significant by two-way ANOVA followed by Bonferroni’s post hoc test (^#^ *p* < 0.05). (**D**) C2C12 cells transfected with scrambled (SCR-) or with specific siRNA for individual LPA receptors were serum-starved prior to be stimulated with 15 µM C1P for 16 h. [^3^H]Thymidine (0.5 μCi/well) was added during the last hour of incubation. Data are mean ± SEM of at least three independent experiments performed in triplicate. [^3^H]Thymidine incorporation in SCR-siRNA transfected cells was 54,265.67 ± 2870.51 dpm, LPA_1_-siRNA transfected cells was 40,903 ± 4714.41 dpm, LPA_2_-siRNA transfected cells was 63,503 ± 5802.95 dpm, LPA_3_-siRNA transfected cells was 41,470.33 ± 5792.39 dpm. The mitogenic effect of C1P was statistically significant by Student’s *t* test (* *p* < 0.05); the effect of LPA_1_ or LPA_3_ down-regulation was statistically significant by two-way ANOVA followed by Bonferroni’s post hoc test (^#^ *p* < 0.05).

C2C12 cells were found to express LPA_1_, LPA_2_ and LPA_3_; LPA_1_ was the dominant receptor subtype expressed while LPA_2_ and LPA_3_ were less represented (Figure S2). Each of these specific LPAR is coupled to different G proteins, including Gq [44]. Interestingly, the pharmacological blockade of LPA_1_ and LPA_3_ receptors by the specific antagonist Ki16425 (2.5 μM) significantly reduced both the activation of ERK1/2 and Akt as well as the mitogenic effect elicited by C1P (Figure 4A,B).

To further confirm these findings, RNA interference technology was used: cell transfection with specific siRNAs strongly down-regulated individual LPA receptor subtypes (Figure S3). As depicted in Figure 4C, down-regulation of LPA_1_ or LPA_3_ significantly diminished C1P-induced ERK1/2 and Akt activation as well as C1P proliferative effect (Figure 4D), while the silencing of LPA_2_ was ineffective (Figure 4C,D). Collectively, these data demonstrate that LPA_1_ as well as LPA_3_ play a crucial role on C1P proliferative effect in myoblasts, supporting the hypothesis that C1P evokes its mitogenic effect in C2C12 myoblasts through the engagement of LPA_1_ and LPA_3_ and the subsequent activation of ERK1/2 and Akt. To corroborate the role of LPA as mediator of C1P action, we first tested whether the lysolipid could stimulate proliferation in myoblasts.

**Figure 5:**
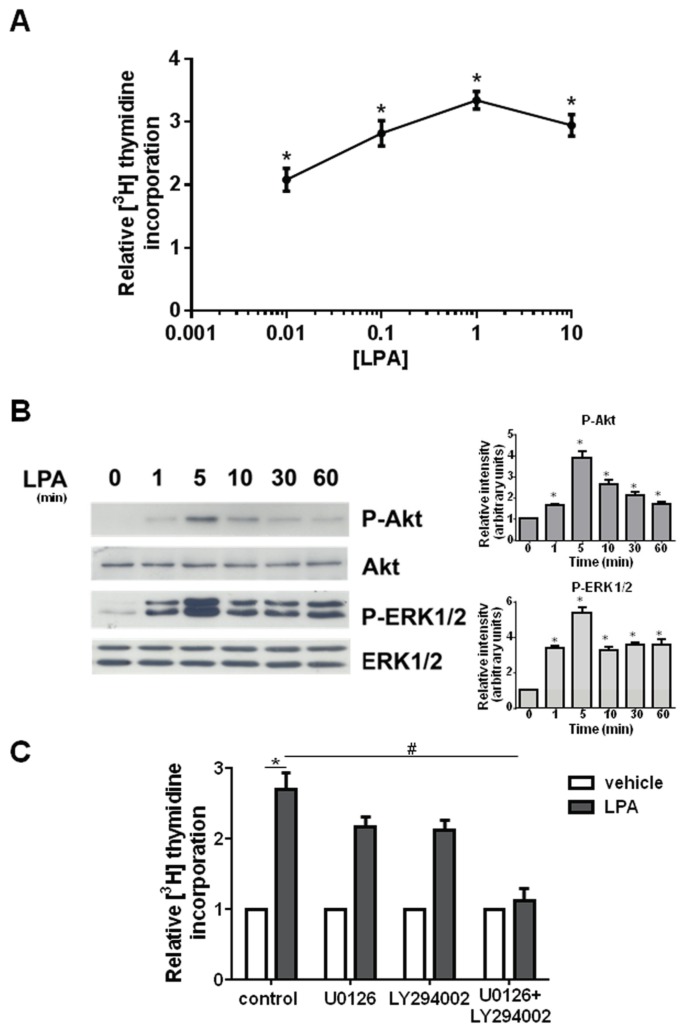
LPA induces cell proliferation via ERK1/2 and Akt in myoblasts. (**A**) Serum-starved C2C12 myoblasts were treated with LPA at the indicated concentrations (0.01–10 μM) for 16 h. [^3^H]Thymidine (0.5 μCi/well) was added during the last hour of incubation. Data are mean ± SEM of at least three independent experiments performed in triplicate. The mitogenic effect of LPA was statistically significant by one-way ANOVA followed by Bonferroni’s post hoc test (* *p* < 0.05). (**B**) Western blot analysis was performed using specific anti-phospho-ERK1/2 and anti-phospho-Akt antibodies in cell lysates prepared from serum-starved myoblasts treated with 100 nM LPA for the indicated time intervals. A blot representative of at least three independent experiments with analogous results is shown. Right panels: Band intensity was quantified by densitometric analysis and normalized to the expression of total ERK1/2 and total Akt, respectively. LPA activates ERK1/2 and Akt in a statistically significant manner by one-way ANOVA followed by Bonferroni’s post hoc test (* *p* < 0.05). (**C**) Serum-starved C2C12 myoblasts were pre-incubated for 30 min in the presence or absence of 5 μM U0126, 5 μM LY294002 or 5 μM U0126 together with 5 μM LY294002 before being stimulated with 100 nM LPA for 16 h. [^3^H]Thymidine (0.5 μCi/well) was added during the last hour of incubation. Data are mean ± SEM of at least three independent experiments performed in triplicate. [^3^H]Thymidine incorporation in untreated control cells was 21,181.25 ± 417.19 dpm, in U0126 treated cells was 6252.67 ± 319.27 dpm, in LY294002 treated cells was 2438.66 ± 221.66 dpm in U0126 and LY294002 treated cells was1594.33 ± 91,30 dpm. The mitogenic effect of LPA was statistically significant by Student’s *t* test (* *p* < 0.05). The effect of both ERK1/2 and Akt inhibition was statistically significant by two-way ANOVA followed by Bonferroni’s post hoc test (^#^ *p* < 0.05).

Results illustrated in Figure 5A clearly show that LPA robustly stimulated DNA replication, determined as [^3^H]Thymidine incorporation into DNA in C2C12 cells. The mitogenic effect of LPA was statistically significant at any of the concentrations tested (0.01–10 µM) and resulted to be concentration dependent.

It was then examined whether LPA could activate the signaling pathways found to be implicated in C1P-induction of cell growth. Data reported in Figure 5B demonstrated that 100 nM LPA potently stimulated ERK1/2 and Akt phosphorylation which peaked at 5 min and remained increased in respect to control up to 60 min of treatment.

To examine the possible role of ERK1/2 and Akt in LPA-mediated myoblast proliferation, [^3^H]Thymidine incorporation experiments were performed in myoblasts previously incubated in the presence of specific pharmacological inhibitors of these pathways. The inhibition of PI3K with 5 µM LY294002 or blockade of ERK1/2 with 5 µM U0126 did not significantly affect the mitogenic action of LPA while the simultaneous inhibition of both signaling pathways abolished the mitogenic response triggered by LPA (Figure 5C). These data suggest that the mitogenic effect exerted by LPA is mediated both by ERK1/2 and Akt activation, each signaling pathway being sufficient for the transduction of the proliferative action of the lysolipid.

**Figure 6:**
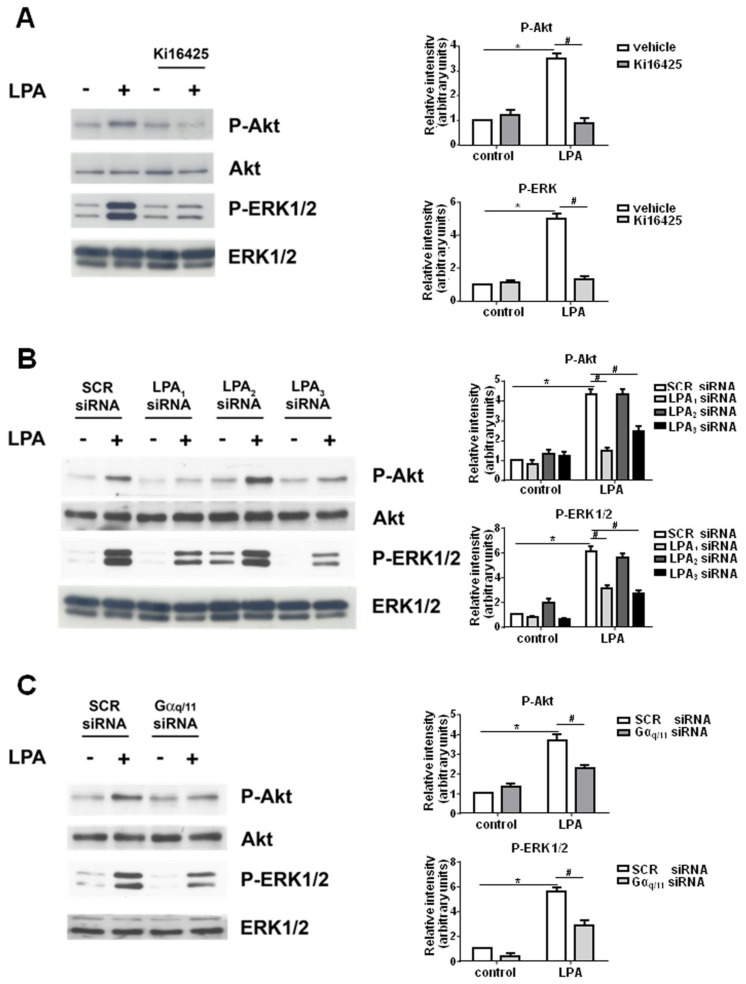
LPA-induced activation of ERK1/2 and Akt requires LPA_1_/LPA_3_ and Gαq/11. Overnight serum-starved C2C12 myoblasts were pre-incubated for 30 min: in the presence or absence of 2.5 μM Ki16425 (**A**); transfected with scrambled (SCR-) or with specific siRNAs for individual LPA receptors (**B**); or transfected with SCR- or Gαq/11-siRNA (**C**), before being stimulated with 100 nM LPA for 5 min. **Left** panels: Western blot analysis performed using specific anti-phospho-ERK1/2 and anti-phospho-Akt antibodies in myoblast cell lysates. A blot representative of three independent experiments is shown. **Right** panels: Band intensity was quantified by densitometric analysis and normalized to the expression of total ERK1/2 and total Akt, respectively. LPA activates ERK1/2 and Akt in a statistically significant manner by Student’s *t* test (* *p* < 0.05); the effect of LPA_1/3_ inhibition by Ki16425 (**A**); the effect of LPA_1_ or LPA_3_ down-regulation (**B**); or the effect of Gαq/11 down-regulation (**C**) was statistically significant by two-way ANOVA followed by Bonferroni’s post hoc test (^#^ *p* < 0.05).

To elucidate the molecular mechanism by which exogenously added LPA exerts its mitogenic action, we analyzed whether the activation of ERK1/2 and Akt relies on LPA_1_ and LPA_3_ engagement. As shown in Figure 6A, the inhibition of LPA_1_ and LPA_3_ by Ki16425 (2.5 μM) blocked the phosphorylation of ERK1/2 and Akt induced by 100 nM LPA for 5 min. In agreement, the LPA-dependent activation of ERK1/2 and Akt was significantly diminished when LPA_1_ or LPA_3_ were down-regulated by RNA interference, whereas was unaffected by LPA_2_ silencing (Figure 6B). Moreover, siRNA-induced down-regulation of Gαq/11 significantly reduced the activation of ERK1/2 and Akt driven by LPA, demonstrating that LPA signaling relies, at least in part, on Gq-coupled receptors (Figure 6C).

**Figure 7:**
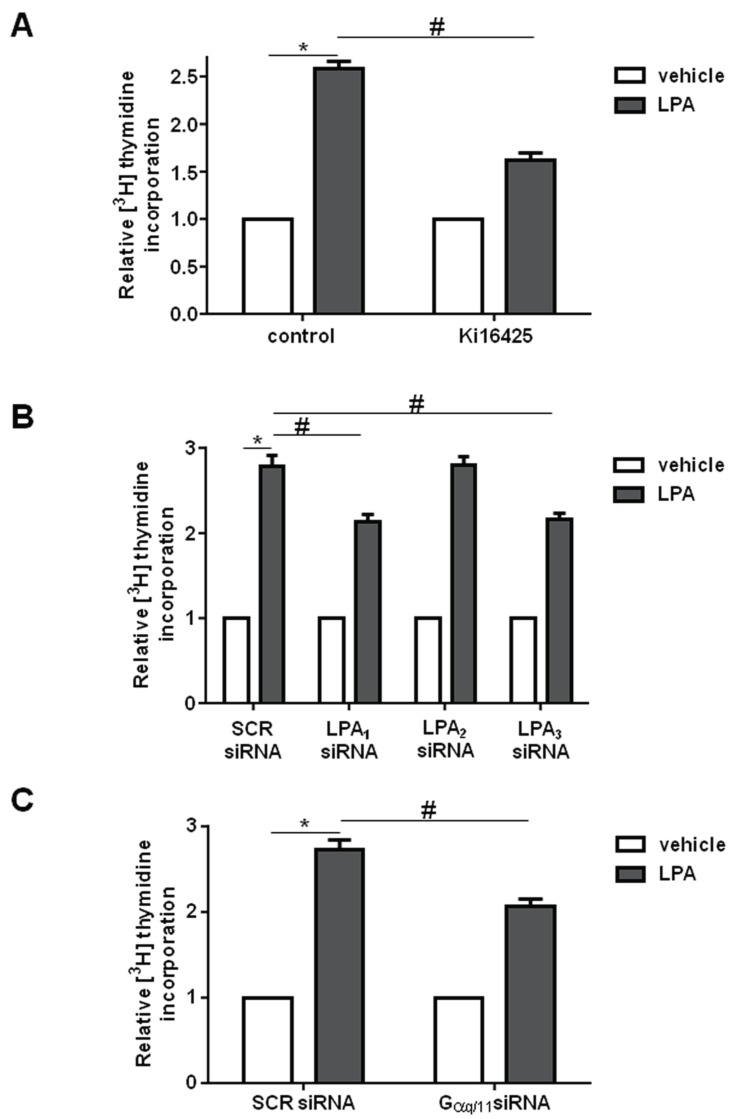
LPA-induced proliferation relies on LPA_1_/LPA_3_ and Gαq/11 engagement. Serum-starved C2C12 myoblasts were pre-incubated for 30 min: in the presence or absence of 2.5 μM Ki16425 (**A**); transfected with scrambled (SCR-) or with specific siRNA for individual LPA receptors (**B**); or transfected with SCR- or Gαq/11-siRNA (**C**) before being stimulated with 100 nM LPA for 16 h. [^3^H]Thymidine (0.5 μCi/well) was added during the last hour of incubation. Data are mean ± SEM of at least three independent experiments performed in triplicate. [^3^H]Thymidine incorporation in untreated control cells was 14,820.5 ± 891.36 dpm, in Ki16425 treated cells was 8959 ± 512 dpm, in SCR-siRNA transfected cells was 54,265.67 ± 2870.51 dpm, LPA_1_-siRNA transfected cells was 40,903 ± 4714.41 dpm, LPA_2_-siRNA transfected cells was 63,503 ± 5802.95 dpm, LPA_3_-siRNA transfected cells was 41,470.33 ± 5792.39 dpm, in SCR-siRNA transfected control cells was 7398 ± 14 dpm, and in Gaq/11 siRNA transfected cells was 1854.33 ± 487.28 dpm. The mitogenic effect of LPA was statistically significant by Student’s *t* test (* *p* < 0.05). The effect of LPA_1/3_ inhibition by Ki16425 (**A**); the effect of down-regulation of LPA_1_ or LPA_3_ (**B**); or the effect of down-regulation of Gαq/11 (**C**) was statistically significant by two-way ANOVA followed by Bonferroni’s post hoc test (^#^ *p* < 0.05).

In accordance with these data, the mitogenic effect of LPA in myoblasts was found to be dependent on the engagement of LPA_1_ and LPA_3_ (Figure 7). Indeed, the enhancement of [^3^H]Thymidine incorporation into DNA elicited by 100 nM LPA was significantly reduced when myoblasts were previously incubated with 2.5 μM Ki16425 (Figure 7A) or when LPA_1_ and LPA_3_ were significantly down-regulated by RNA interference (Figure 7B). Furthermore, the proliferative action of exogenously added LPA was significantly reduced by specific down-regulation of Gαq/11 (Figure 7C) supporting a key role of Gq-mediated signaling in the proliferative effect of the bioactive lipid.

**Figure 8:**
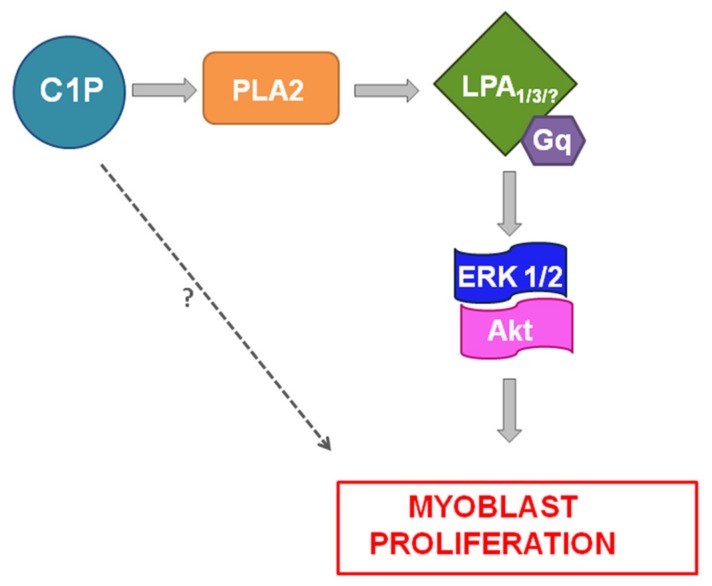
C1P stimulates C2C12 myoblast proliferation. The dashed arrow and question marks indicate unexplored pathways that could be partially implicated in C1P mitogenic action in myoblasts.

In summary, the here obtained results demonstrate that C1P exerts its mitogenic effect through LPA_1_ and LPA_3_ engagement which drive ERK1/2 and Akt activation thus stimulating myoblast proliferation (Figure 8).

## 3. Discussion

Skeletal muscle is a post-mitotic tissue composed of myofibers that accounts for 30–50% of body mass in humans. The tissue retains the ability to regenerate thanks to the presence of resident muscle stem cells named satellite cells capable of self-renewing and differentiating into myoblasts, which then fuse one to each other to form myofibers. The niche where satellite cells reside is composed of myofibers, extracellular matrix proteins, macrophages and regulatory T-cells that form a multifaceted microenvironment crucial for an effective regenerative response following skeletal muscle trauma [25]. Since muscle as well as non-myogenic cells secrete growth factors and cytokines that influence satellite cell behavior, the full comprehension of the molecular mechanisms by which diverse signals ensure the complete potential of skeletal muscle precursors is the ultimate goal for therapeutic approaches aimed at restoring skeletal muscle function.

The bioactive sphingolipid C1P has recently emerged as a positive cue for muscle regeneration since it has been shown to significantly stimulate skeletal muscle cell proliferation [9]. Here, we identified a totally novel mechanism of action by which C1P stimulates myoblast proliferation. Namely, C1P via PLA2 activation drives the engagement of LPAR, LPA_1_/LPA_3_, which are in turn responsible for Akt and ERK1/2 activation and cell proliferation induced by the bioactive sphingolipid.

The mitogenic action of C1P has been reported for different cell types such as fibroblasts [12] and macrophages [10]. Interestingly, C1P stimulated proliferation of fibroblasts was found to be independent on ERK1/2 activation as well as c-myc or c-fos expression [12], while in quiescent primary macrophages, in agreement with our findings, the mitogenic action of C1P was mediated by ERK1/2 and Akt activation [10]. Moreover, proliferation of lung adenocarcinoma cells induced by EGF has been shown to rely on CerK, responsible for C1P generation, which subsequently leads to ERK1/2 and Akt activation [45].

Our findings enlarge the knowledge on the crucial role of bioactive sphingolipid metabolism and signaling, vital for skeletal muscle function. In particular, the proliferative action of C1P in myoblasts appear to be precise and distinct from that of other sphingolipids. For example, S1P has been reported to exert a pro-myogenic and an anti-proliferative effect in myoblasts being able of reducing the mitogenic action of serum [28], while ceramide [35,36] and sphingosine [46] have been shown to inhibit myogenic differentiation.

Interestingly, treatment with PTx did not influence C1P-induced myoblast proliferation ruling out the involvement of a Gi coupled C1P receptor in myoblasts [9], differently from macrophages, where C1P-induced migration was shown to depend on the presence of a specific Gi-coupled C1P receptor that caused phosphorylation of ERK1/2 and Akt [15]. Here, the possible involvement of C1P specific receptors coupled to G proteins different from Gi was excluded by performing C1P binding experiments in C2C12 myoblasts.

However, the fast time-course of ERK1/2 and Akt activation induced by C1P, compatible with receptor-mediated events, and the involvement of Gq proteins in the observed biological effect, forced to look for alternative explanations. Inhibition of PLA2 by MAFP demonstrated that this enzyme is required for C1P induced proliferation in myoblasts, confirming the well-established role of C1P as activator of PLA2 both in vitro and in vivo [42,43]. Nevertheless, inhibition of COX1 and COX2 by pretreatment with Indomethacin, Rofecoxib as well as SC-236 proved that prostanoid generation from arachidonate generated by PLA2 action was not involved in C1P mediated myoblasts proliferation. Intriguingly, the here reported mechanism of action by which C1P exerts its proliferative effect supports the notion that C1P, in addition of playing a crucial role as mediator of inflammatory reactions [47] can regulate diverse fundamental cellular processes. Whether C1P-induced PLA2 activation in myoblasts, besides driving mitogenesis, is involved in inflammatory responses remains to be clarified.

PLA2 hydrolyzes the sn-2 (sn-1) ester bond of phosphatidic acid (PA) to generate LPA. LPA is not only a metabolite involved in the synthesis of membrane phospholipids but also a crucial bioactive cue capable of influencing a broad variety of biological processes such as growth, survival, chemoresistance by binding to G protein-coupled receptors [48]. Different PLA2 isoforms display an exclusive or relative selectivity for PA [49], however, the contribution of each PLA2 to LPA synthesis is not precisely known. cPLA2 is localized in the soluble fraction of the cell and translocates to membranes as consequence of activation. Whether C1P activates cPLA2 directly [18,43] or via PKC [17] as shown in other cell contexts, and where exactly this signaling occurs within the cell, remains to be investigated. Recently, intracellular targets for LPA have been highlighted; LPA was indeed shown to bind to the nuclear hormone receptor peroxisome proliferator-activated receptor that regulates genes involved in energy metabolism control [50].

LPAR couple to members of three major G protein families, the Gi, Gq and G12 family [44]. C2C12 myoblasts express LPA receptors [51]; LPA_1_ and LPA_2_ are known to interact with all three G protein families while LPA_3_ interacts with Gi and Gq, but not with G12 proteins; and LPA_4_ also couple the Gs family [44]. Since the here presented data showed that the effect of C1P was incompletely blocked by Ki16425, the involvement of other LPAR expressed in myoblasts cannot be presently excluded. It has been previously reported that LPA phosphorylates ERK1/2 and Akt in C2C12 myoblasts thus activating mitogenic cascade [51]. The here reported findings support a role also for LPAR coupled to Gq proteins since the blockade of Gq signaling significantly reduced thymidine incorporation as well as ERK1/2 and Akt phosphorylation induced by LPA.

Unfortunately, attempts to measure increased levels of LPA following C1P treatment either inside the cells or in the extracellular medium by LS/MS mass spectrometry failed, at least at the investigated time points, leaving open alternative molecular mechanisms other than LPA signaling axis instrumental for C1P action. Very limited information is available in the literature on the time-dependence of LPA production inside the cells in comparison to abundant reported findings on autotaxin-mediated generation of extracellular LPA [52,53]. Consistently, LPA levels in cellular membranes are very low [54] since LPA is expected to be rapidly metabolized by the action of LPA acyltransferase, which convert LPA back to PA, and by PA phosphohydrolases and lysophospholipases that rapidly degrade the lysophospholipid [54].

Questions also arise on whether and how LPA, intracellularly produced, is released outside the cell. Little is known about how this transport would happen compared for example to the export of the bioactive sphingolipid S1P through ABC transporters or the specific transporter Spinster homologue 2 (SPNS2) [55]. LPA levels are elevated in plasma from oncologic patients: phorbol 12-myristate 13-acetate stimulates LPA secretion from different cancer cell lines in a dose and time-dependent manner only when cultured in the presence of serum, suggesting that tumor cells do not produce LPA when growth factors are depleted [56].

Here, for the first time, we provide evidence that the bioactive sphingolipid C1P uses the signaling axis of another pleiotropic and structurally simple lipid, LPA, to exploit its action. Cross-talk between LPA and sphingolipid signaling has been previously reported: we demonstrated that the migratory effect of LPA in murine skeletal muscle activated satellite cells requires S1P signaling axis since when both SK isoforms, SK1 and SK2, and S1P_1_/S1P_4_ were blocked, the pro-migratory action of LPA was significantly reduced [34]. Moreover, it has been shown that the proliferative action of LPA in cancer gastric cells is mediated by the SK1 isoform upregulation via activation of the LPA_1_ [57] and that down-regulation of SK1 attenuated LPA-stimulated migration and invasion of MKN1 gastric cells.

Our results shed new light on the key role of bioactive sphingolipids in skeletal muscle and provide further support to the notion that these pleiotropic molecules might be useful therapeutic targets for skeletal muscle regeneration.

## 4. Materials and Methods

### 4.1. Materials

All biochemicals, TRI reagent, cell culture reagents, Dulbecco’s Modified Eagle Medium (DMEM), fetal bovine serum, protease inhibitor cocktail, bovine serum albumin (BSA), Ki16425, Rofecoxib, Indomethacin and natural C1P (from bovine brain, containing predominantly stearic and nervonic acids) were purchased from Sigma-Aldrich (St. Louis, MO, USA). 1-Oleoyl Lysophosphatidic Acid (LPA), Methyl Arachidonyl Fluorophosphonate (MAFP) and SC-236 were purchased from Cayman Chemical (Ann Arbor, MI, USA). Mouse skeletal muscle C2C12 cells were obtained from the American Type Culture Collection (Manassas, VA, USA). LY294002 hydrochloride and U0126 were from Tocris Cookson Limited (Bristol, UK). Phospho-ERK1/2 and pan ERK1/2 antibodies were from Cell Signaling Technology, Inc. (Beverly, MA, USA). siRNA duplexes were obtained from Sigma-Proligo (The Woodlands, TX, USA). Lipofectamine RNAiMAX was purchased from Invitrogen (Carlsbad, CA, USA). Enhanced chemiluminescence reagents was obtained from GE Healthcare Europe (Milan, Italy). Pan-Akt (H-136), phospho-pan-Akt (Ser 473), monoclonal anti-β-actin, anti-COX2 and anti-PKCα antibodies as well as secondary antibodies conjugated to horseradish peroxidase were obtained from Santa Cruz Biotechnology (Santa Cruz, CA, USA). All reagents and probes required to perform real-time PCR were from Applied Biosystems (Foster City, CA, USA). [^3^H]Thymidine (20 Ci/mmol) was from Perkin Elmer (Waltham, MA, USA). [^3^H]C1P (Ceramide-d-erythro-1-phosphate [*N*-stearoyl-9,10-^3^H]) was purchased from American Radiolabeled Chemicals, Inc. (ARC) Saint Louis, MO, USA.

### 4.2. Cell Culture

Murine C2C12 myoblasts were routinely grown in DMEM supplemented with 10% fetal bovine serum, 2 mM l-glutamine, 100 U/mL penicillin, and 100 μg/mL streptomycin at 37 °C in 5% CO_2_. For the experiments, cells were seeded and, when approximately 50% confluent, they were serum-starved in DMEM without serum containing 1 mg/mL BSA. When requested, cells were incubated with inhibitors 30 min before challenge with agonists.

### 4.3. Cell Transfection

Cell transfection was performed using Lipofectamine RNAiMAX according to the manufacturer’s instructions as previously reported [30,58]. Experiments were performed within 48 h from the beginning of transfection. For Gαq/11 inhibition, C2C12 cells were transiently transfected with pcDNA3.1-RGS3CT [38] or pRK5-GqI [39]) vector using Lipofectamine 2000 reagent (1 mg/mL), as described previously [31].

### 4.4. Cellular Fractionation

C2C12 cells were washed twice with ice-cold PBS and scraped in 10 mM HEPES, pH 7.4, 1 mM EDTA, 1 mM EGTA, 250 mM sucrose, 5 mM NaN_3_ and protease inhibitors (1 mM 4-(2-aminoethyl) benzenesulfonyl fluoride, (AEBSF), 10 μg/mL leupeptin, 10 μg/mL pepstatin, and 0.3 μM aprotinin). Lysates were achieved disrupting the cells using a Dounce homogenizer (100 strokes). Cytosolic and total particulate fractions were obtained by centrifugation as previously described [30,33].

### 4.5. Western Blot Analysis

To prepare total cell lysates, C2C12 cells were incubated for 30 min at 4 °C in 50 mM Tris, pH 7.5, 120 mM NaCl, 6 mM EGTA, 1 mM EDTA, 20 mM NaF, 15 mM Na_4_P_2_O_7_, 1% Nonidet and protease inhibitor cocktail (1.04 mM AEBSF, 0.02 mM leupeptin, 0.08 μM aprotinin, 15 μM pepstatin A, 0.04 mM bestatin and 14 μM E-64) before being centrifuged for 15 min at 10,000× *g* at 4 °C. Samples resuspended in Laemmli’s SDS (sodium dodecyl sulphate) sample buffer were subjected to SDS-PAGE before transfer of proteins to PVDF (polyvinylidene difluoride) membranes. Membranes were incubated overnight with the primary antibodies at 4 °C and then with secondary antibodies for 1 h at room temperature. Chemiluminescence was used to detect bound antibodies.

### 4.6. Cell Proliferation

Cell proliferation was determined by [^3^H]Thymidine incorporation; C2C12 cells were serum-starved for 24 h and then challenged with or without 15 µM C1P or different concentration of LPA for 16 h. After [^3^H]Thymidine (0.5 μCi/well) addition in the last 1 h of incubation, cells were washed twice in ice-cold PBS and then 500 μL of 10% trichloroacetic acid were added. Successively, cells were washed in ice-cold PBS, before being added with 250 μL of ethanol:ether (3:1 *v*/*v*) and lysed in 0.25 N NaOH for 1 h, as previously described [30]. [^3^H]Thymidine incorporation was measured by scintillation counting.

### 4.7. Cell Membrane Preparation for C1P Radioligand-Binding Assay

C2C12 cells were plated onto 100-mm diameter dishes at 1 × 10^5^ cells/dish and were grown in DMEM containing 10% FBS. The cells were incubated in homogenization buffer (10 mM Tris–HCl, 3 mM EDTA, 3 mM EGTA, 1 mM NaF, pH 7.5) containing 1 μL/mL protease inhibitor cocktail and 1 mM phenylmethylsulfonyl fluoride (PMSF) for 30 min on ice. They were then lysed with a Dounce homogenizer and the remaining intact cells and nuclei were removed by centrifugation at 500× *g* for 5 min. Cell membranes were pelleted by centrifugation at 100,000× *g* for 30 min and resuspended in binding buffer (50 mM Tris–HCl, 150 mM NaCl, 0.8% fatty acid-free BSA, 1 μL/mL protease inhibitor cocktail, and 0.2 mM PMSF, at pH 7.5). Only freshly prepared membranes were used in experiments. [^3^H]C1P (final specific activity 60 nCi/pmol) and non-labeled C1P were sonicated in fatty acid-free BSA binding buffer and mixed with membranes in a total volume of 150 µl in borosilicate tubes. Binding was performed at 37 °C with gentle mixing for 30 min, and terminated by collecting the membranes onto GF/C filter with a 1225 Sampling Manifold from Millipore. To determine the non-specific binding of the radioligand to the filters, the same experiment was performed as described above but without cell membranes. Filters were then rapidly washed three times with 350 μL of ice-cold washing buffer containing 10 mM Tris–HCl, and 15 mM NaCl, at pH 7.5 with a 1225 Sampling Manifold from Millipore. Radioactivity of filter-bound radionuclide was quantified by liquid scintillation counting.

### 4.8. Quantitative Real-Time Reverse Transcription PCR

C2C12 myoblasts total RNA was extracted with TRI reagent, before being reverse transcribed by the high capacity cDNA reverse transcriptase (Applied Biosystems). Relative quantitative real-time PCR was performed using TaqMan gene expression assays to quantify LPAR mRNA. The automated ABI Prism 7500 Sequence Detector System (Applied Biosystems) was employed as previously described [59,60,61], simultaneously amplifying the target sequence together with the housekeeping gene 18 S rRNA. Results were analyzed by ABI Prism Sequence Detection System software, version 1.7 (Applied Biosystems). The 2^−ΔΔ*C*t^ method was applied as a comparative method of quantification [62], and data were normalized to ribosomal 18 S RNA expression.

### 4.9. Statistical Analysis

To perform densitometric analysis of the Western blot bands and graphical representations, ImageJ software and GraphPad Prism 6.0 (GraphPad Software, San Diego, CA, USA) were utilized, respectively. Statistical analysis was performed using Student’s *t* test, one-way ANOVA and two-way ANOVA followed by Bonferroni’s post hoc test (* *p* < 0.05, # *p* < 0.05).

## Supplementary Materials

Supplementary materials can be found at www.mdpi.com/1422-0067/19/1/138/s1.

## Abbreviations

LPA	Lysophosphatidic acid
C1P	Ceramide 1-phosphate
COX	Cyclooxygenase
Cerk	Ceramide kinase
PLA2	Phospholipase A2
LPAR	Lysophosphatidic acid receptor
