# Supplementary material for: Lysophosphatidic Acid Signaling Axis Mediates Ceramide 1-Phosphate-Induced Proliferation of C2C12 Myoblasts
Source: Int J Mol Sci. 2018 Jan 4;19(1):139. doi: 10.3390/ijms19010139 (PMC5796088; doi:10.3390/ijms19010139)
Supplement: Supplementary file 1 [file ijms-19-00139-s001.pdf]

Supplementary Information

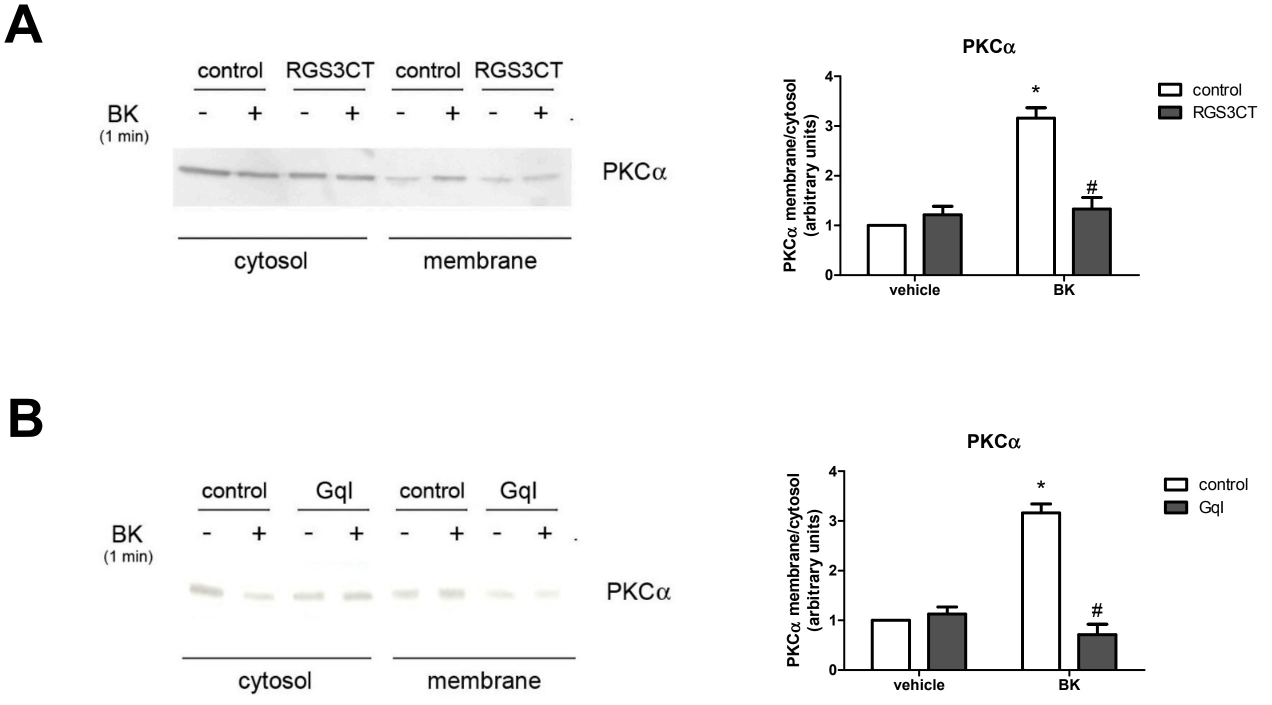

Figure S1. Bradykinin-induced PKCα translocation to membrane fraction relies on G$\alpha_{q/11}$ engagement.

C2C12 cells were transiently transfected with pcDNA3.1-RGS3CT or empty vector (control) (**A**) or with pRK5-GqI or empty vector (control) (**B**). Cells were overnight serum-starved prior to be stimulated with 1 μM bradykinin (BK) for 1 min. Left panels: PKCα activation was evaluated as translocation of the enzyme to the membrane. Western blot analysis of PKCα were performed in membrane and cytosolic fractions. Blots representative of at least three independent experiments are shown. Right panels: the histograms represent densitometric analysis of three independent experiments. Data reported are expressed as fold increase of the membrane:cytosol ratio. The increase of PKCα membrane content induced by BK was statistically significant by Student's t test (*p<0.05); the effect of G$\alpha_{q/11}$ inhibition on BK-induced PKCα activation was statistically significant by two-way ANOVA followed by Bonferroni's post hoc test (#p<0.05).

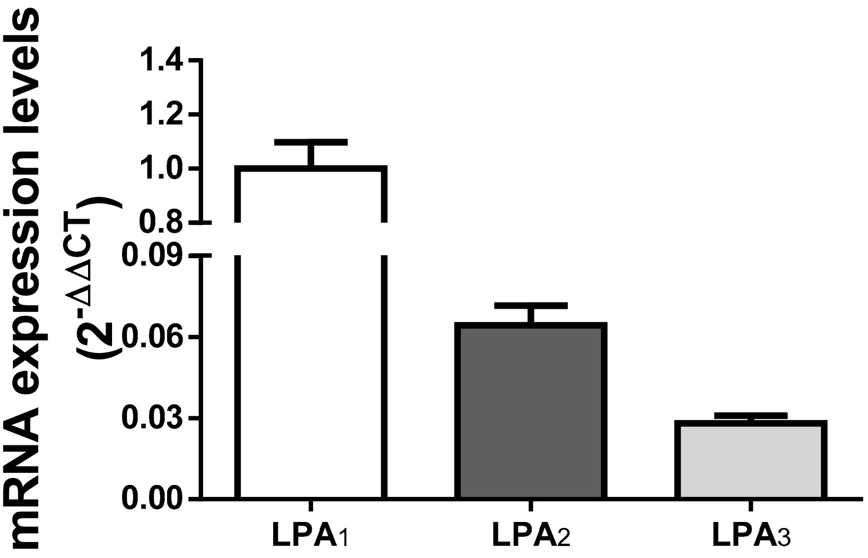

Figure S2. Expression of LPA receptors at the mRNA level.

Quantitative mRNA analysis was performed by real-time PCR by concurrent amplification of the target sequence of murine LPA_1, LPA_2 and LPA_3 genes together with that of 18S rRNA. The results are expressed as fold changes according to the $2^{-\Delta\Delta CT}$ method, using LPA_1 as calibrator. Values are means±SEM of three independent experiments performed in triplicate.

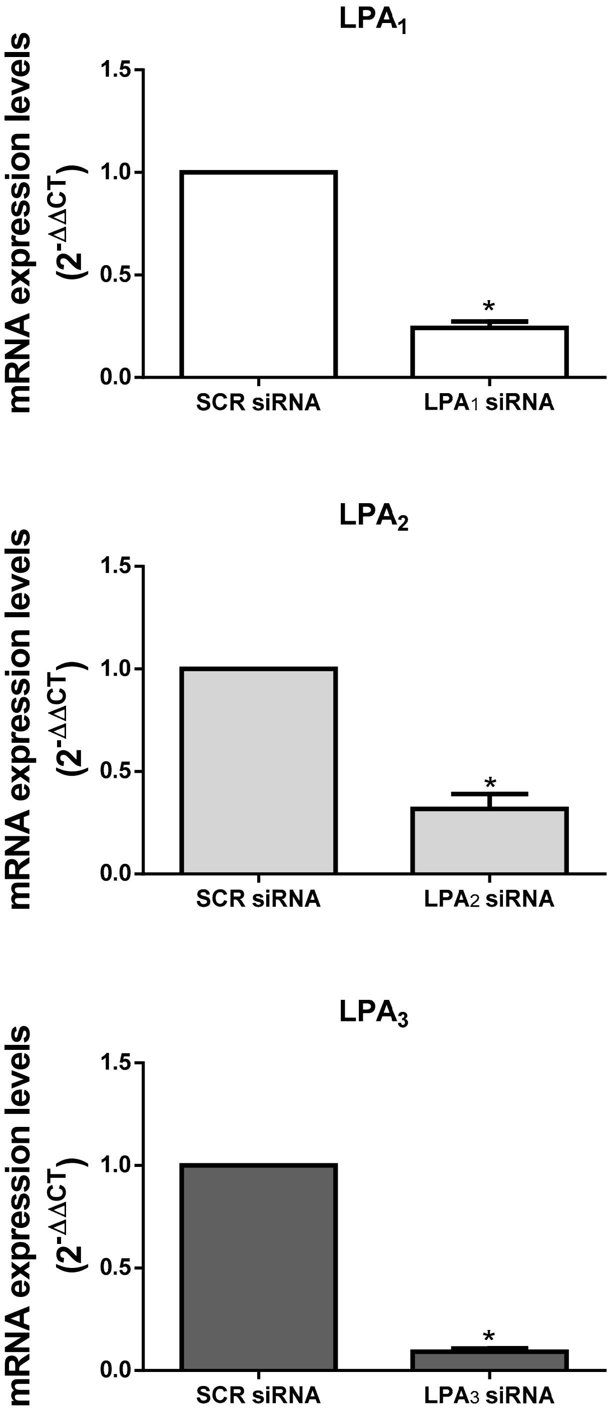

Figure S3. Down-regulation of LPAR.

C2C12 myoblasts, transfected with scrambled (SCR) or with specific siRNA for individual LPA receptors, were checked for downregulation by real-time PCR by concurrent amplification of the target sequence of murine LPA_1, LPA_2 and LPA_3 genes together with that of 18S rRNA. The results are expressed as fold changes according to the $2^{-\Delta\Delta CT}$ method. Values are means±SEM of three independent experiments performed in triplicate. The effect of siRNA transfection on LPAR expression is statistically significant by Student's *t* test (*$p<0.05$).
